# Pattern of peripapillary capillary density loss in ischemic optic neuropathy compared to that in primary open-angle glaucoma

**DOI:** 10.1371/journal.pone.0189237

**Published:** 2018-01-10

**Authors:** Masoud Aghsaei Fard, Yanin Suwan, Sasan Moghimi, Lawrence S. Geyman, Toco Y. Chui, Richard B. Rosen, Robert Ritch

**Affiliations:** 1 Farabi Eye Hospital, Tehran University of Medical Science, Tehran, Iran; 2 Department of Ophthalmology, Ramathibodi Hospital, Mahidol University, Bangkok, Thailand; 3 Einhorn Clinical Research Center, New York Eye and Ear Infirmary of Mount Sinai, New York, NY, United States of America; 4 Icahn School of Medicine at Mount Sinai, New York, NY, United States of America; Bascom Palmer Eye Institute, UNITED STATES

## Abstract

**Purpose:**

Both non-arteritic anterior ischemic optic neuropathy (NAION) and primary open-angle glaucoma (POAG) damage retinal ganglion cell axons, which are perfused by the radial peripapillary capillaries. To evaluate the pattern of ischemia, we compared peripapillary capillary density (PCD) in NAION eyes to POAG eyes matched for visual field mean deviation and retinal nerve fiber layer thickness.

**Methods:**

31 chronic NAION (>6 months after the acute event) and unaffected fellow eyes (31 subjects), 42 moderate and severe POAG eyes (27 subjects), and 77 control eyes (46 healthy subjects) were imaged with a commercial optical coherence tomography angiography system (AngioVue, Avanti RTVue-XR, Optovue, CA) at two academic institutions. Two concentric circles of diameters 1.95mm (inner) and 3.45mm (outer) were manually placed on images centered on the optic nerve head, producing an annular region-of-interest. Image analysis with major vessel removal was performed using a custom program. Whole-image, whole-annulus, and sectoral PCDs were measured.

**Results:**

Whole-image and whole-annulus PCDs in NAION and moderate and severe POAG eyes were significantly decreased compared to unaffected fellow eyes and control eyes (all P<0.001). Superior and temporal PCD values were affected more than other sectors in both NAION and POAG groups compared to control group. Whole-image and whole-annulus PCDs were not statistically different between NAION and POAG eyes (both P = 0.99). However, of all peripapillary sectors, the inferior sector PCD value was less affected in POAG eyes compared to NAION eyes (P = 0.001). Univariate analysis results also revealed a significant positive correlation between superior and inferior PCDs and corresponding RNFL thicknesses. The inferior sector correlation was greater in POAG than NAION eyes.

**Conclusion:**

While the whole PCD values were not different in chronic NAION and POAG, the greater correlation of inferior PCD with corresponding RNFL sectors in POAG compared to NAION suggests greater susceptibility of the inferior radial peripapillary capillary in the pathogenesis of POAG.

## Introduction

While nonarteritic anterior ischemic optic neuropathy (NAION) is the clinical presentation of acute ischemic damage to the optic nerve, primary open–angle glaucoma (POAG) is characterized by chronic progressive retinal ganglion cell (RGC) death and axon loss [1, 2].

Various methods have been used to measureoptic nerve head (ONH) perfusion in NAION and POAG, many focusing on the peripapillary capillaries, a superficial capillary bed within the retinal nerve fiber layer (RNFL) that supplies the RGC axons [3–14]. Both Laser Doppler flowmetry and Laser Speckle Flowgraphy have demonstrated ONH head perfusion defects in both glaucomatous and ischemic optic neuropathies [3, 4]. Prior studies have demonstrated the capability of a novel imaging modality, optical coherence tomography angiography (OCT-A), to provide aquantitative assessment of the circulation in the ONH and peripapillary vasculature [5–7] and have identified a decrease in the peripapillary vessel density in eyes with POAG and NAION compared to controls [5–7, 10–14].

We have previously shown that there exist distinct patterns of damage to the RGCs in POAG compared to NAION eyes, with a greater loss of inner RGC regions (parafovea) in the NAION eyes [2]. Therefore, the patterns of radial peripapillary capillaries (RPC) supplying the RGC axons may reflect these differences in damage. In addition, although vessel density calculations in several prior studies included the contribution of large peripapillary vessels [6, 11, 12, 14]. We believe that such measures do not accurately represent the microvasculature (RPC), and, as such, we employed a custom algorithm to exclude the major vessels in our measurement of the peripapillary capillary density (PCD) [8].

In this study, in order to evaluate the pattern of optic nerve ischemia, we compared PCD in NAION eyes to that in POAG eyes with moderate and severe damages, matched for visual field mean deviation (MD) and RNFL thickness.We also investigated the correlations between PCD and RNFL thicknesses in these two optic neuropathies.

## Materials and methods

### Subjects

Patients with chronic (post-acute) unilateral NAION and moderate and severe POAG and healthy control subjects who were seenat the New York Eye and Ear Infirmary of Mount Sinai and Farabi Eye Hospital between February 2016 and May 2016 were enrolled in this cross-sectional, comparative study. The study was approved by the New York Eye and Ear Infirmary of Mount Sinai Institutional Review Board and Ethics Committee of Tehran University of Medical Science and all investigations adhered to the tenets of the Declaration of Helsinki. Written informed consent was obtained from each participant after receiving a detailed explanation of the nature and objective of the study.

#### Control group

The control group included age-matched subjects with a best-corrected visual acuity ≥20/30, intraocular pressure (IOP) ≤21 mm Hg, an open angle, normal optic disc appearance on fundus examination, and no visual field or RNFL defects.

In all groups, patients below 18 years of age and those with refractive errors ≥+6.00 or ≤-6.00 D or more than ±3.00 D astigmatism, a history of ocular surgery (except for uncomplicated cataract surgery), or a glaucomatous or neurological disease were also excluded.

#### Unilateral NAION

Inclusion criteria for chronic unilateral NAION included: a history of sudden, painless visual loss in one eye together with optic disc swelling and/or superficial hemorrhage on the border of the disc or adjacent retina typical of NAION that occurred>6 months earlier (by confirming the objective signs or by asking the referring ophthalmologists), plus an ophthalmologically healthy fellow eye. At the time of the study, in the affected eye, optic disc borders were sharp and discrete, and the optic disc swelling had subsided. Patients having or suspected of havingan ocular or neurologic disease other than NAION, including but not limited tosuspected glaucoma, bilateral NAION, acute NAION, arteritic AION and/or inflammatory optic neuritis were excluded from this study.

#### Moderate and severe POAG

Patients withPOAG manifested the following: enlargement of the vertical cup-to-disc ratio, apparent difference in the vertical cup-to-disc ratio between both eyes (more than 0.2), and diffuse or focal thinning of the neuroretinal rim, and an open iridocorneal angle on gonioscopy. The Hodapp-Parrish-Anderson criteria were used for the diagnosis and staging of glaucomatous visual fields [15]. A glaucomatous visual field was defined as (1) a glaucoma hemifield test (GHT) outside normal limits on at least two consecutive baseline visual field tests and (2) the presence of at least three contiguous test points within the same hemifield on the PSD plot at p<1%, with at least one at p<0.5%, excluding points on the edge of the field or those directly above and below the blind spot. The two baseline tests required reliability indices better than 25% in order to be included. To make glaucoma and NAION groups more similar in terms of severity, only patients with mean deviation (MD) scores less than -6 dB (moderate and severe POAGs) were recruited in this study[2]. Patients with ahistory of ocular surgery other than uncomplicated cataract surgery; a history of ocular or neurologic disease other than glaucoma; and/or a history of secondary glaucoma, angle closure glaucoma, or early glaucoma were excluded.

### Methods

All subjects underwent a thorough ophthalmic evaluation, including best-corrected visual acuity (BCVA), slit-lamp biomicroscopy, IOP by Goldmann applanation tonometry, dilated fundus examination, gonioscopy, and axial length measurement (IOLMaster, Carl Zeiss Meditec, Dublin, California, USA). Perimetry was performed using the standard 24–2 Swedish Interactive Thresholding Algorithm (SITA) on the Humphrey Field Analyzer (Carl Zeiss Meditec, Dublin, CA). Only reliable results were included (fixation losses<20%, false-positive errors<15%, and false-negative errors<15%(. Patients then underwent OCT and OCT-A imaging.

#### OCT circumpapillary retinal nerve fiber layer analysis

Optical coherence tomography (Spectralis OCT Version 6.0.11.0; Heidelberg Engineering, GmbH, Germany) was performed prior to OCT-A to acquire global (average) and sectoral circumpapillary RNFL thickness values. Images with poor centration, segmentation errors, or poor quality (<15 decibels) were excluded from analysis.

#### Optical coherence tomography angiography

For OCT-A, we used a commercial spectral domain-OCT system (AngioVue Software Version 2016.1.0.90, Optovue, Fremont, CA, USA) that employs the split spectrum amplitude-decorrelation angiography (SSADA) algorithm [5]. The system uses an 840-nm light source and an A-scan rate of 70,000 scans/sec in a 4.5×4.5 mm rectangle scan centered on the ONH. In order to produce images of the vasculature, the system analyzes the vessels located between the internal limiting membrane (ILM) and the posterior boundary of the RNFL using AngioVue software. Patients with poor image quality including images with poor clarity, motion artifact, and scans with signal strength index of <40 were excluded.

#### OCT-A image analysis

OCT-A images were analyzed using a custom MATLAB program (The Mathworks, Inc., Natick, MA, USA) [8, 16] Each grayscale was resized by a factor of six and contrast stretched by using the lowest and highest 1% of pixel intensity values of the image as the lower and upper limits, respectively. Global thresholding was performed to convert the grayscale OCTA image into a binary image by replacing all pixel intensities>0.55 with the value of 1 (white) and the remaining pixels with the value of 0 (black). After the removal of large blood vessels from the binary image, local adaptive thresholding was performed using a sampling window size of 15x15 pixels, in order to account for local differences in brightness throughout the image.

**Fig 1:**
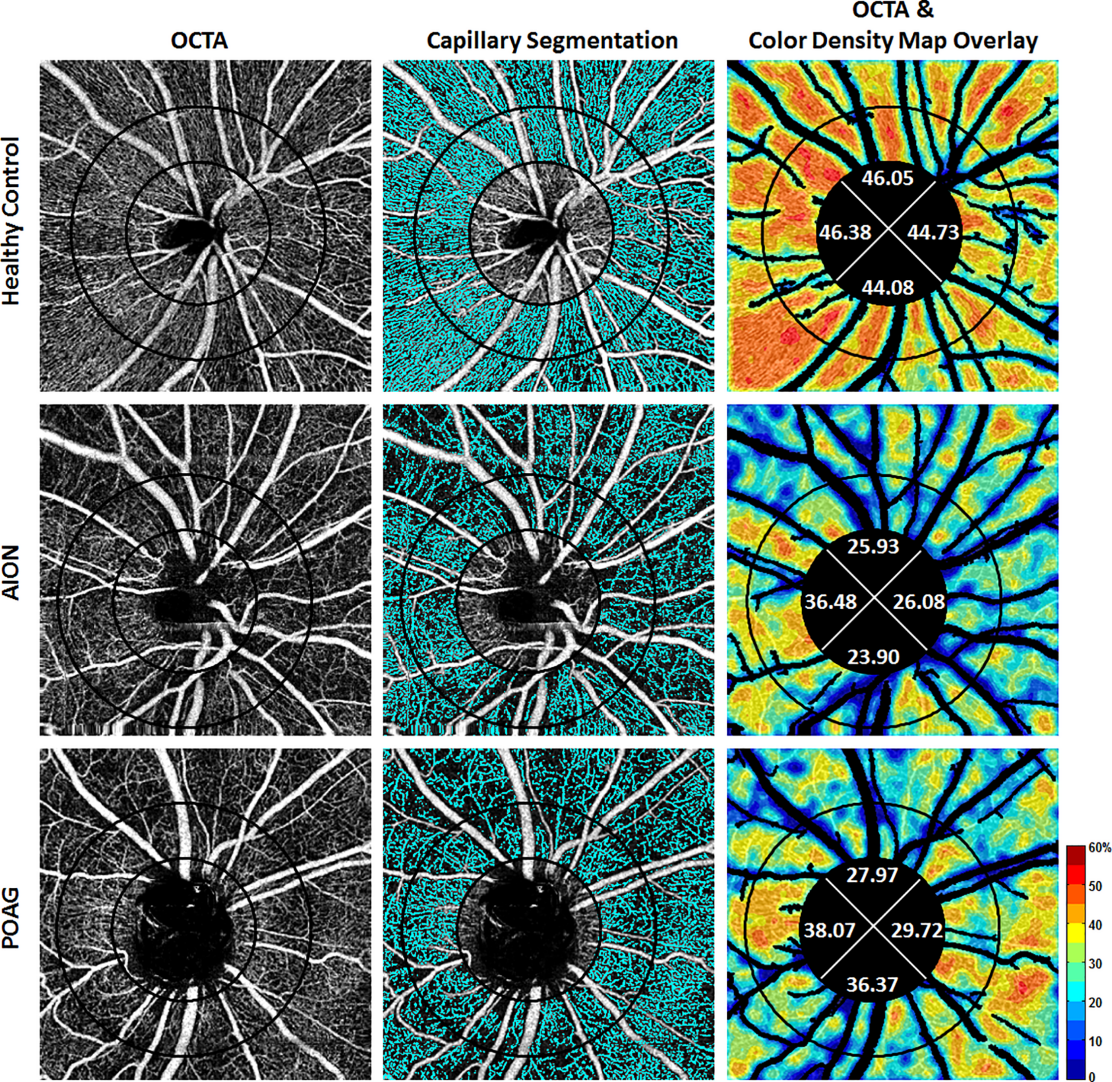
Peripapillary capillary density maps. Comparisons of peripapillary capillary density maps in a normal, an anterior ischemic optic neuropathy (AION) eye with average retinal nerve fiber layer (RNFL) thickness of 54 μm and visual field mean deviation of -20.5 dB, and a severe primary open-angle glaucoma (POAG) eye with average RNFL thickness of 49 μm and mean deviation of -25.2 dB. *First column*: grayscale OCT-A images with two concentric circles with 1.95-mm (inner) and 3.45-mm (outer) diameters, *Second column*: Perfused capillary area (in *cyan*) after the removal of major blood vessels. *Third column*: corresponding color-coded perfused capillary density maps with numerical capillary density (%) in peripapillary ring in four quadrants.

Whole PCD was calculated as a percentage by dividing the number of pixels associated with perfused capillaries over the number of pixels in the entire image after removal of the inner 1.95-mm circular area and the major blood vessels. To calculate whole-annulus and sectoral PCD, a 3.45mm-diameter outer circle was placed concentric to the inner 1.95-mm circle, producing an annular region of interest (ROI) with a width of 0.75mm. This 3.45mm outer circle diameter parallels the dimensions of the standard RNFL thickness circle scan currently employed in the majority of commercially available OCT systems. To ensure that the same ROI was included in all OCTA images, PCD within a fixed annular ROI was extracted for quantitative analysis. PCD was then calculated as a percentage by dividing the area associated with perfused capillaries (white pixels) over the area of the ROI-in-question (either the entire annulus or one of the four 90-degree sectors). A color-coded PCD map that was generated by computing the PCD within a 16x16-pixel sampling window with 8-pixel overlaps over the binary image (Fig 1).

### Statistical analysis

The distribution of numerical data was tested for normality using the Shapiro-Wilk test. Descriptive statistics were calculated as the mean and standard deviation for normally distributed variables. Categorical variables were compared using the Chi-squared test. We also employed a linear mixed model to evaluate the differences of PCD and RNFL parameters between groups using the Bonferroni correction for multiple comparisons and inter-eye correlation after adjusting for age, gender, and axial length. Finally, we compared PCD sector differences within each group using repeated measure analysis.

Univariable linear regression models were built using PCD (annular and sectoral) as the dependent variable and RNFL thickness (global and sectoral) and visual field (MD) as the independent variables in each group (control and POAG eyes, NAION and unaffected eyes). To evaluate the differences between the correlations of the PCD with RNFL in two sectors within groups, we used the package ‘cocor’ in R (R Core Team (2014). R: A language and environment for statistical computing. R Foundation for Statistical Computing, Vienna, Austria. URL http://www.R-project.org/). All other statistical analyses were performed with the SPSS software (IBM Corp. Released 2013. IBM SPSS Statistics for Windows, Version 22.0. Armonk, NY: IBM Corp.). P-values <0.05 were considered significant.

## Results

Thirty-three NAION subjects (66 eyes), 29 POAG subjects (45 eyes), and 49 control subjects (80 eyes).Two NAION, three normal, and 2 POAG subjects were excluded because of poor signal quality, dense cataract, or eye movement. As a result, 31 chronic unilateral NAION and their unaffected fellow eyes (31 subjects), 42 POAG eyes (12 moderate POAG and 30 severe POAG eyes of 27 subjects), and 77 control eyes (46 subjects) were analyzed.

Statistically significant differences were found between the NAION eye and the contralateral unaffected eyes in terms of visual acuity (logMAR), MD, global RNFL thickness, and sectoral RNFL thickness (for all sectors). Similar significant differences were found between POAG eyes and control eyes. Visual field MD and average RNFL were not statistically different between NAION and POAG eyes (Table 1).

**Table 1:** Demographic and ocular characteristics of healthy, anterior ischemic optic neuropathy and unaffected fellow eyes, and moderate-to-severe primary open-angle glaucoma patients.

	NAION	Fellow Eye	POAG	Control	*P1*	*P2*	*P3*
	(n = 31)	(n = 31)	(n = 42)	(n = 77)			
**Age, yrs.**	54.1±11	-	60.2±8	58.4±10.3	-	>0.99	0.07
**Gender, female:male**	15:16	-	16:26	42:35	-	0.08	0.38
**Axial length, mm**	22.4±1.5	22.2±1.8	23.2±1.3	23±1.4	>0.99	>0.99	0.23
**Visual Acuity, LogMAR**	0.71±0.73	0.08±0.13	0.29±0.33	0.09±0.12	<0.001	0.02	<0.001
**Mean deviation, dB**	-18.4±8.6	-1.0±2.7	-17.2±8.6	-0.6±1.7	<0.001	<0.001	>0.99
**Average RNFL,μm**	59.8±16.3	102.2±10.5	64.0±14.1	98.2±10.3	<0.001	<0.001	0.95
**Superior RNFL,μm**	57.8±16.1	126.4±19.1	77.6±22.4	121.3±20.4	<0.001	<0.001	0.001
**Nasal RNFL,μm**	52.7±23.1	80.5±15.4	59.0±15.1	74.0±11.3	<0.001	<0.001	0.58
**Inferior RNFL,μm**	69.7±21.3	133.1±17.3	66.3±17.4	127.0±17.4	<0.001	<0.001	>0.99
**Temporal RNFL,μm**	48.4±26.5	68.1±13.1	52.2±14.6	68.8±12.4	<0.001	<0.001	>0.99

RNFL, retinal nerve fiber layer; P1, comparison of AION (anterior ischemic optic neuropathy) & fellow eyes; P2, comparison of POAG (primary open-angle glaucoma) & control; P3, comparison of POAG & AION eyes using linear mixed model.

A linear mixed model, adjusted for age, gender, axial length and inter-eye correlation, demonstrated that the PCD values were significantly different among the groups (P <0.001 for both whole-image PCD and whole-annular PCD). POAG and NAION eyes had significantly lower whole-image and whole-annulus PCD (30.2±5.1% and 31.3±5.6%) when compared with control eyes and unaffected fellow eyes, respectively (all P<0.001). Whole-image and whole-annulus PCD were not statistically different between NAION and POAG eyes (Both P = 0.99) (Table 2).

**Table 2:** Mean and standard deviation values for OCT-A peripapillary capillary density measurements in healthy participants, primary open-angle glaucoma, and anterior ischemic optic neuropathy patients and their unaffected fellow eyes.

	NAION	Fellow Eye	POAG	Control	*P1*	*P2*	*P3*
**Whole-PCD,%**	30.1±6	41.6±4.5	30.2±5.1	42.3±2.3	<0.001	<0.001	>0.99
**Whole-annular PCD,%**	29.7±6.5	42.3±5	31.3±5.6	43.9±2.0	<0.001	<0.001	0.22
**Superior PCD,%**	26.8±9.3	41.5±7.0	29.1±7.6	43.7±2.2	<0.001	<0.001	0.63
**Nasal PCD,%**	32.5±6.8	42.9±4.3	32.2±7.9	43.3±2.7	<0.001	<0.001	>0.99
**Inferior PCD,%**	31.2±7.6	42.6±4.7	36.1±6.9	43.9±2.2	<0.001	<0.001	0.001
**Temporal PCD,%**	28.2±8.4	42.5±4.9	28.0±8.2	44.6±2.2	<0.001	<0.001	>0.99

PCD, peripapillary capillary density; P1, comparison of AION (anterior ischemic optic neuropathy) & fellow eyes; P2, comparison of POAG (primary open-angle glaucoma) & control; P3, comparison of POAG & AION eyes using linear mixed model.

In the NAION group, superior PCD had lower values than nasal and inferior sectors (P<0.001 and P = 0.001) and temporal PCD was lower than nasal PCD (P = 0.03). In POAG group, there was a significant decline in superior and temporal PCDs compared to inferior sector (both P<0.001). When comparing POAG and NAION groups, of all peripapillary sectors, inferior sector PCD value was significantly lower in NAION eyes compared to POAG eyes (P = 0.001).

Univariate regression analysis showed that correlation between whole-image PCD and visual field MD was significant both in POAG group (r = 0.82, P<0.001) and NAION group (r = 0.69, P<0.001). Results from the regression analysis for PCD and RNFL thickness in NAION and POAG groups are summarized in Table 3. In both groups superior and inferior sector correlations with corresponding RNFL thickness were more than correlations of temporal and nasal sectors (Table 4). Correlation between inferior PCD and inferior RNFL thickness in POAG and control subjects were more than the same feature in NAION and their fellow eyes. (P = 0.032)

**Table 3:** Association between different variables using univariate analysis in study groups.

Correlated variables in study groups	R^2^	95% CI for B coefficient	*P value*
**POAG and control subjects**			
Superior-annular PCD, Superior RNFL	0.57	2.20–3.05	<0.001
Nasal-annular PCD, nasal RNFL	0.18	0.50–1.16	<0.001
Inferior-annular PCD, inferior RNFL	0.46	3.12–4.70	<0.001
Temporal-annular PCD, temporal RNFL	0.22	0.49–1.02	<0.001
**NAION and unaffected fellow eyes**			
Superior-annular PCD, Superior RNFL	0.45	1.70–3.03	<0.001
Nasal-annular PCD, nasal RNFL	0.15	0.47–1.95	<0.001
Inferior-annular PCD, inferior RNFL	0.36	1.73–3.54	<0.001
Temporal-annular PCD, temporal RNFL	0.16	0.39–1.48	<0.001

PCD, peripapillary capillary density; RNFL, retinal nerve fiber layer; AION, anterior ischemic optic neuropathy; POAG, primary open angle glaucoma.

**Table 4:** Differences of the correlations of capillary density and nerve fiber layer in two sectors in two groups of subjects.

Two sectors with correlated PCD and corresponding RNFL thickness	Difference of correlations of two sectors	P value of difference
**POAG and control subjects**		
Superior versus nasal sectors	0.33	<0.001
Superior versus inferior sectors	0.08	0.126
Superior versus temporal sectors	0.29	<0.001
Inferior versus nasal sectors	0.25	0.001
Inferior versus temporal sectors	0.21	0.004
Temporal versus nasal sectors	0.04	0.637
**NAION and unaffected fellow eyes**		
Superior versus nasal sectors	0.28	0.003
Superior versus inferior sectors	0.05	0.218
Superior versus temporal sectors	0.26	0.017
Inferior versus nasal sectors	0.23	0.046
Inferior versus temporal sectors	0.21	0.105
Temporal versus nasal sectors	0.02	0.896

PCD, peripapillary capillary density; RNFL, retinal nerve fiber layer; AION, anterior ischemic optic neuropathy; POAG, primary open angle glaucoma. P value using ‘cocor’ in R.

## Discussion

We evaluated the pattern of PCD loss in NAION eyes compared to POAG eyes matched for visual field defects and RNFL thickness using OCTA. In both NAION and POAG eyes, superior and temporal PCD were affected more than nasal and inferior sectors. Whole-image PCD and annular PCD were not different in chronic NAION and POAG. Of all peripapillary sectors, inferior sector PCD value was significantly higher in POAG eyes compared to NAION eyes. Univariate analysis results also revealed a significant positive correlation between superior and inferior PCDs and corresponding RNFL thicknesses. Inferior sector correlations were greater in POAG than NAION eyes.

Vascular dysfunction has been proposed to play an important role in glaucomatous optic neuropathy, and clinical studies have shown decreased peripapillary vessel density in glaucomatous eyes [11, 12, 16–18]. OCT-A studies also have shown optic nerve blood flow abnormalities in NAION [13, 14]. Recent study found a decrease in peripapillary vessel density (including large vessels and small capillaries) at the corresponding location of visual field defects in NAION using OCT-A software’s intensity-based thresholding technique [14]. In addition, similar to our study, they found significant correlations between RNFL thickness and the vessel density in the superior and inferior regions in NAION eye.

In this study POAG and NAION eyes had significantly lower whole-image and whole-annulus PCD when compared with control eyes and unaffected fellow eyes, respectively. Greater involvement of superior and temporal PCDs was observed both in NAION and glaucoma eyes. It has been demonstrated that ganglion cell damage in NAION is more noticeable in the inner nasal and superior macula, [2] which were supplied by superior and temporal PCD. Although in POAG, the nasal macula and temporal optic nerve is often preserved, we observed significant loss of temporal PCD. Because we enrolled moderate and severe POAG cases, in which temporal disc would be involved, we also observed temporal PCD loss. In addition, as the correlation between temporal PCD and temporal RNFL is not as strong as superior and inferior sectors, temporal PCD loss might occur earlier than temporal RNFL loss. In fact, in progressive glaucomatous damage, vascular microcirculation defects in temporal RPC may precede structural changes in the retinal nerve fiber layer [19].

We also compared PCD in POAG and NAION. Although optic nerve ischemia occurs in both conditions, the role of vascular insult seems to be more relevant in NAION than glaucoma. One early study had found greater proximal vasoconstriction (narrowed retinal arteries near the disc) using optic disc photographs in NAION than in glaucoma [20]. Interestingly, degrees of whole and annular PCD loss in these two forms of optic nerve damage were not different. It has been shown in glaucoma that greater PCD loss occurs with greater RNFL loss [12]. Therefore, we selected moderate and severe POAG cases with matched RNFL thickness. In our study whole peripapillary vessel density were affected similar to average RNFL in both NAION and POAG independent of optic nerve damage. It was recently suggested that the dropout of RPCs is not specific to POAG or NAION, and it might be secondary to RGC damage [14]. Lee et al. [21] also proposed that the decreased density of retinal microvasculature probably represents the closure or degeneration of capillaries that occurs along with the RNFL loss rather than primary reduction of retinal perfusion. In contrast, a recent study showed greater decrease in the peripapillary vessel density in the NAION group than the moderate POAG eyes using OCT-A software which was explained by less severe attenuation of peripapillary retinal vasculature in moderate-stage POAG instead of severe glaucoma [22]. However, their sample size was smaller than our study and was not statistically normally distributed. In addition, they measured peripapillary large and small vessels density instead of peripapillary microvascular.

It was also observed in this study that the correlations between inferior PCD and inferior RNFL sectors in POAG eyes were generally stronger than the correlations in NAION eyes. Several studies have highlighted the importance of RPC networks, which supplied with central retinal artery in glaucoma using OCT-A studies. Actually, RPC performs as well as RNFL thickness for discriminating between healthy and glaucoma patients and for differentiating between the healthy and glaucoma suspect groups [6]. In fact, the inferior RNFL had the highest diagnostic ability for distinguishing eyes with glaucomatous from controls and the inferior optic nerve head had greater susceptibility to glaucomatous damage [23, 24]. On the other hand, the primary site of optic nerve head lesion in NAION is mainly nourished by the microcirculation of the posterior ciliary artery [1] and the primary ischemia in NAION is not at the level of the RPCs. Then, loss of RPC in NAION eyes in this study seems due to secondary RNFL loss. Therefore, inferior sectors vascular-structural correlation in NAION was less than the same measure in POAG.

This study has several other limitations. First, the RNFL and the PCD are calculated using two different instruments, and the correspondence of the sectors could be not accurate. Second, capillaries skeletonization was not performed during image processing. Third, measuring vessel caliber of OCT-A images after removing large vessel with our software is not easy to do and may not be accurate and this point could be a focus of future studies. Lastly, evaluating the temporal relationship between PCD and RNFL damage needs longitudinal study and the cross-sectional design of this study has limitations and determination of cause-consequence relationship between peripapillary capillary and RNFL remain unclarified. Additional work is required to address this issue.

### Conclusion

Whole-image PCD and annular PCD were affected to similar degrees in chronic (post-acute) NAION and moderate-to-severe POAG and there was a strong correlation between peripapillary capillary morphology and RNFL thickness.

In both NAION and POAG eyes, superior and temporal PCD were affected more than nasal and inferior sectors. Correlations between inferior PCD and RNFL sectors in POAG eyes were generally stronger than the correlations in NAION eyes.

## Supporting information

S1 File Patients data in each group. (0: control group; 1: Ischemic optic neuropathy group; 2: contralateral unaffected eye of ischemic optic neuropathy eyes; 4: open angle glaucoma group).
(XLSX)
